# AI in imaging: the regulatory landscape

**DOI:** 10.1093/bjr/tqae002

**Published:** 2024-01-04

**Authors:** Derek L G Hill

**Affiliations:** UCL, Gower Street, London, WC1E 6BT, United Kingdom

**Keywords:** radiological, AI, machine learning, medical device, regulation, bias

## Abstract

Artificial intelligence (AI) methods have been applied to medical imaging for several decades, but in the last few years, the number of publications and the number of AI-enabled medical devices coming on the market have significantly increased. While some AI-enabled approaches are proving very valuable, systematic reviews of the AI imaging field identify significant weaknesses in a significant proportion of the literature. Medical device regulators have recently become more proactive in publishing guidance documents and recognizing standards that will require that the development and validation of AI-enabled medical devices need to be more rigorous than required for tradition “rule-based” software. In particular, developers are required to better identify and mitigate risks (such as bias) that arise in AI-enabled devices, and to ensure that the devices are validated in a realistic clinical setting to ensure their output is clinically meaningful. While this evolving regulatory landscape will mean that device developers will take longer to bring novel AI-based medical imaging devices to market, such additional rigour is necessary to address existing weaknesses in the field and ensure that patients and healthcare professionals can trust AI-enabled devices. There would also be benefits in the academic community taking into account this regulatory framework, to improve the quality of the literature and make it easier for academically developed AI tools to make the transition to medical devices that impact healthcare.

## Introduction

Machine learning and artificial intelligence (AI) methods have been applied to medical imaging applications for several decades, with publications and dedicated conferences on the topic in the 1990s.^1–3^ In recent years, however, there has been a rapid acceleration in activity in this area, both in academic research and in the launch of commercial products. AI is achieving an ever-higher profile in the mass media, most recently with the high-profile launch of several Generative AI tools. The general public is increasingly aware of the potential impact of AI and machine learning on their lives, and of the benefits and risks of AI, and this may be especially the case where AI impacts their health.

Because AI imaging tools have applications in the diagnosis and management of patients, they come under the definition of medical devices, and the medical device regulators are therefore key gatekeepers in the arrival of such AI tools on the market. AI applications in medical imaging are now entering the healthcare market in significant numbers. The US Food and Drug Administration (FDA), which currently has the most comprehensive database of medical devices, periodically publishes the number of AI-enabled medical devices that have received market authorizations (eg, 510k clearance, de novo). A review of devices cleared between 2019 and 2021 was published by Muehlematter et al.^4^

The most recent FDA publication, published October 19, 2023,^5^ reports that, up to the end of July 2023, a total of 692 AI-enabled medical devices had received marketing authorization, of which more than 75% are for radiology applications.

The regulators need to strike a balance between enabling innovation in this important area and ensuring that AI tools put on the market have a positive benefit: risk ratio for patients. This article looks at applications of AI in medical imaging, the evolving regulatory landscape for AI-enabled medical devices, and the implications of this for the developers and users of AI medical imaging applications, and the medical imaging community more broadly.

## State of the art of AI in medical imaging

Given this article focuses on the regulatory landscape for AI in medical imaging, it is appropriate to start with a medical device regulators definition, recently published by the FDA.^6^ We will use this definition throughout the rest of this publication, and we will use “artificial intelligence” (AI) as shorthand for this definition.

*Artificial Intelligence (AI) and Machine Learning (ML) can be described as a branch of computer science, statistics, and engineering that uses algorithms or models to perform tasks and exhibit behaviors such as learning, making decisions, and making predictions. ML is considered a subset of AI that allows models to be developed by training algorithms through analysis of data, without models being explicitly programmed.*

Barragan-Montero et al, in a thorough technical review,^7^ emphasize that the most recent innovation in AI in medical imaging has been in that machine learning subset of AI. In machine learning, an algorithm learns from data without needing to be explicitly programmed with a set of rules. The types of algorithms we are considering in this article are those based on *machine learning*, as distinct from *rule-based* approaches.

In general, the medical images used for training AI models have been pre-labelled. This labelling is very often done by experts, who delineate image features by hand. For example, an algorithm to find the boundary of the left ventricle in a cardiac ultrasound scan may be trained with images that have been carefully delineated by a radiologist or ultrasound technician using a drawing tool on a workstation. However, labelling may also be done based on data that are not in the images; for example, an algorithm might be trained to tell the difference between patients with rapidly progressing or slowly progressing disease by training with longitudinal outcome data.

One particular type of machine learning, referred to as deep learning, has recently become extremely widespread in medical imaging applications. Deep learning is used to describe methods in which more sophisticated AI models, typically multiple-layer neural networks, are trained to classify input data. They have been shown to provide better performance than more traditional data-driven machine learning methods. Barragan-Montero et al’s review provides a helpful summary of the various deep learning approaches and highlights that convolution neural networks are currently viewed as state of the art for many medical imaging applications.

Recently, generative AI has received a lot of publicity because of its use in natural language processing, and the launch of generative AI applications such as OpenAI’s ChatGPT based on large language models. Such applications can generate content that may be indistinguishable from human-generated content and has been shown to be able to pass third-year medical student exams.^8^

Gong et al^9^ have reviewed the application of Generative AI in medical imaging, and its use to create models trained on existing medical image data to generate new images with similar underlying properties to real-world images. While made-up medical images might provide opportunities for fraud in medical research, it is not yet clear how these might be relevant to patient management. Gong et al, however, point out that they do provide a way of generating large volumes of training images for training traditional deep learning algorithms, from a smaller set of hand-annotated images. Generative AI may, therefore, be seen as a solution to the challenge of obtaining sufficient labelled images to train AI algorithms for medical applications. We will return to this topic later in the review, as it highlights the challenge of obtaining truly representative images for training and testing.

Regardless of the underlying algorithmic approach, there are a wide variety of applications of AI in medical imaging that are proposed in the literature. This variety of applications can be illustrated by dividing AI applications into the following five categories, based on the output they generate and the way this output is then used in a clinical setting:

1. *Automatic delineation of a structure of interest* in an image, where that structure is known to be present. This structure might be one or more chambers of the heart from MRI,^10^ a liver from abdominal CT,^11^ the hippocampus from volumetric MRI.^12^
2. *Detecting abnormalities* in a medical image, for example, detecting lesions in mammograms,^13^ or glioma in MRI.^14^ Unlike the first category, this approach involves detecting whether an abnormality is present in the image, rather than measuring a structure that is known to be present.
3. *Image Enhancement* such as improving the resolution of reconstructed images such as deep learning super-resolution, for example, to reduce examination time while preserving image diagnostic quality.^15^
4. *Identifying a disease-specific signature*, learned from multiple image features, that could be used in the diagnosis of a disease, for example, an imaging signature of Alzheimer disease (AD) from MRI.^16^ Unlike categories 1 and 2, this application of AI goes beyond automating a task that could be done by a radiologist on a workstation. These approaches may use information from sources other than just the images to generate their output.
5. *Predicting outcomes* based on medical images, for example, predicting outcomes from ischaemic stroke,^17^ intracranial aneurysm rupture, in COVID-19,^18^ or in oncology.^19^ These approaches may also use information from sources other than just the images to generate their output.

These five categories of imaging AI could be considered to be in order of increasing potential to positively impact healthcare: the first two categories involve automating a task that might currently be performed by a radiologist. The third category uses AI to provide radiologists with higher-quality images than would otherwise have been available. The fourth and fifth categories, however, are more disruptive as they provide information that could not be obtained from a traditional radiology read, and such approaches might be used to streamline clinical workflows, with AI review of images being used to directly impact patient management decisions without the input of a radiologist.

Within all these categories, it is possible to have AI algorithms that are trained once, validated, and then used (“train-then-validate”), or algorithms that, once they enter clinical use, re-train themselves using additional data received (“continuous learning”). The “train-then-validate” approach is more consistent with the traditional way that medical device software is written, with the software developed, “frozen”, and then validated, with the validated device then submitted for regulatory review prior to being put on the market. Any significant subsequent update in the software would require a re-review of the technical file. The “continuous learning” approaches do not fit into this traditional medical device framework. While they provide a means to continuously improve the performance of AI tools once they enter clinical use, safeguards would be needed to ensure that the performance does not worsen with additional training, and in particular, the algorithms do not get better at generating incorrect output. As a consequence, continuously learning algorithms are at a much earlier stage in terms of impacting medical practice.

A fundamental concept in considering the application of AI in imaging is the assessment of the performance of the AI tool in a realistic clinical environment.

## Evaluating the performance of AI-based medical imaging applications

There are increasing numbers of publications in the academic literature that assess the performance of AI methods in medical imaging, and report that AI methods can perform as well or better than normal clinical practice. Examples include breast cancer detection in mammography^20^ and stroke.^21^

However, there are also publications that point out the limitations of AI methods and find that radiologists outperform AI tools. A study comparing four commercially marketed AI tools for assessment of chest radiographs found concerning rates of false positives.^22^

During the COVID-19 pandemic, there was very rapid implementation and publication of AI-based methods to try to help manage patients, including through predicting outcomes from chest CT scans. Wynants et al’s review of these publications^18^ reported that most were poorly reported and at high risk of bias, therefore potentially of very limited clinical value.

The volume of academic literature on AI in medical imaging is vast, with tens of thousands of papers being published per year,^7^ but systematic reviews of these approaches often exclude the great majority for methodological reasons, suggesting only a small fraction of the work in this area may be close to clinical applicability.^23^ There are increasing numbers of papers that highlight the risks that many AI products may not be sufficiently safe and effective for clinical use, and encourage the development of suitable “comprehensive guidelines for their implementation”.^24^

This demonstrates the importance of considering how the performance of AI imaging tools should be evaluated both before, and after, they are put on the market. It is quite possible that the performance an AI tool achieves while under development is not replicated when it enters clinical use.

The main challenge facing the field is often identified as the difficulty in obtaining sufficient high-quality labelled data to train the AI models. In breast imaging, for example, it has been shown that algorithms perform best when trained with large volumes of highly annotated data, with key image features all meticulously labelled by an expert annotation.^20^ There are two distinct phases in developing an AI tool: *training* and then *testing*. These tasks are distinct and make use of *training* data and *test* data, respectively. While there are huge numbers of medical images collected each day in clinical practice, the need for high-quality annotations means that image data for training and testing algorithms are often in short supply. As a result, developers frequently use the same dataset for both training and testing, using a method called “cross-validation”. A common approach is to divide the dataset into *k* equal-sized subsets, then repeat the train-test cycle *k* times, in each case using a different subset for testing, and the remaining *k*-1 subsets for training. Performance is assessed by averaging the performance over the *k* repeats. In this approach, the data used for training is different from the data used for testing, but it is not truly *independent*, as all the training and test data come from the same original dataset. The alternative approach is to train the algorithm on one dataset, and test it on an independently acquired dataset, for example, collected from different hospitals or over a different time period. The systematic review by Borchert et al^25^ reported that “studies using an independent dataset for validation, as opposed to cross-validation, reported much lower accuracy particularly when community-based population was used”. This has led many to question whether cross-validation provides a reliable measure of the performance of AI tools in clinical practice.

Medical device regulators, aware of this literature and of concerns around the performance of products on the market, have been busy over recent years responding to the rapid increase in AI applications by providing additional clarity on how medical device regulations apply to AI-enabled devices.

## The AI regulatory landscape

Medical device *regulations* do not specifically deal with the use of AI in medical devices, often because the regulations were put in place before AI became widely used. The regulatory landscape is therefore defined by those regulations that apply to software, augmented by publications from regulators, such as guidance documents, discussion documents, and recognized standards that help manufacturers apply the medical device regulations to their products.

From a medical device regulatory point of view, the amount of performance data required to show that a medical device is safe and effective is dependent on what the manufacturer claims the device can do. These claims are captured not only in the device’s specific regulatory documentation as intended use, intended purpose, or instructions for use, but also in associated marketing material, whether in hard copy, on the website, or in social media posts, all of which are considered by regulators to be part of the device “labelling”. Earlier in this article, we illustrated 5 different categories of AI applications that illustrate the variety of intended use: the type and amount of performance data required to show adequate performance will clearly vary. Rapid innovation in AI applications has resulted in increasing interest from medical device regulators, and in some cases, significant changes in the documentation required before AI imaging tools can be put on the market.

A particular feature of software medical devices, as distinct from traditional “hardware” medical devices such as a joint implant, is that the performance of the device can be significantly changed by upgrading the software, which can be undertaken much more rapidly than updating the design and performance of a hardware medical device, and with the upgrade deployed entirely remotely. And unlike traditional hardware devices, software upgrades could also change the intended use of the device, for example, from providing a radiologist with decision support through prompting for lesions in a radiograph, to finding the lesions automatically and generating a treatment plan, with no input from a radiologist.

Medical device regulators worked together under the auspices of the International Medical Device Regulatory Form (IMDRF) to publish guidance on the Clinical Evaluation of Software as a Medical Device (SaMD) that provided a risk framework and guidance on validation, for SaMD.^26^ This introduces important concepts for manufacturers, including the “clinical association” between device output and the targeted clinical condition, as part of the clinical evaluation process required before putting the device on the market (reproduced in Table 1), and a two-dimensional risk-categorization framework that takes account both of the significance of the information provided by the software on the healthcare decision, and the state of healthcare situation or condition (reproduced in Table 2). While this guidance applies to machine learning software, it does not treat it in a fundamentally different way from other medical device software.

**Table 1.** Clinical evaluation process.

Valid clinical association	Analytical validation	Clinical validation
Is there a valid clinical association between your SaMD output and your SaMD’s targeted clinical condition?	Does your SaMD correctly process input data to generate accurate, reliable, and precise output data?	Does use of your SaMD’s accurate, reliable, and precise output data achieve your intended purpose in your target population in the context of clinical care?

**Table 2.** SaMD risk categories intended medical purpose (horizontal) vs targeted healthcare condition (vertical).

State of healthcare situation or condition	Significance of information provided by SaMD to the healthcare decision		
	Treat or diagnose	Drive clinical management	Inform clinical management
Critical	IV	III	II
Serious	III	II	I
Nonserious	II	I	I

The rapid evolution of AI means that regulators are increasingly wanting to treat AI differently from traditional software, and we are therefore seeing regular publications from regulators focused on AI-enabled devices.

In a further example of collaboration between medical device regulators, the US FDA, UK MHRA, and Health Canada jointly published “Good Machine Learning Practice: guiding principles” in October 2021.^27^ This is a short document that captures some aspects of good practice in the development of medical devices that incorporate machine learning. Table 3 reproduces these guiding principles.

**Table 3.** Good machine learning practice for medical device development: guiding principles.

Multi-disciplinary expertise is leveraged throughout the total product life cycle	Good software engineering and security practices are implemented
Clinical study participants and datasets are representative of the intended patient population	Training datasets are independent of test sets
Selected reference datasets are based upon available methods	Model design is tailored to the available data and reflects the intended use of the device
Focus is placed on the performance of the Human-AI team	Testing demonstrates device performance during clinically relevant conditions
Users are provided clear and essential information	Deployed models are monitored for performance and re-training risks are managed

While these guiding principles are helpful, for example, stating that independent test data (rather than cross-validated methods) should be used, it is not in all cases clear how to show compliance. In order to provide greater clarity to developers, the FDA recognized as a “consensus standard”, a guidance document published by AAMI CR34971:2022 for the application of the established medical device risk management standard, ISO14971, to medical devices incorporating AI and machine learning. This document has subsequently been released by BSI as BS/AAMI 34971:2023, demonstrating its international impact. This publication starts with a cautionary note:

*Despite the sophistication and complicated methodologies employed, machine learning systems can introduce risks to safety by learning incorrectly, making wrong inferences, and then recommending or initiating actions that, instead of better outcomes, can lead to harm.*

*The amplification of errors in an AI system has the potential to create large scale harm to patients.*

*With medical devices without AI, risk can be assessed from real-world experience with that technology. With AI-enabled medical devices, however, that experience is lacking …. it may be more complex to identify risks and bias since the algorithmic decision pathways may be challenging to interpret.*

Risk management in medical devices is already focused on possible harm to patients and the hazardous situation that can give rise to that harm. This AAMI publication highlights the fact that AI introduces new possible hazards that are not properly covered by current product development methodology for “rule-based” algorithms, and provides a detailed recipe for how to handle risk in AI software. Table 4 gives the risks highlighted in this document.

**Table 4.** Risk categories for AI/ML medical devices, to be incorporated in ISO14971 risk analysis.

Data quality	Bias	Data storage/security/privacy	Overtrust
Incorrect data	Selection bias	Privacy failures	Overconfidence
Incomplete data	Confounding variables	Bias due to privacy	Perceived risk
Subjective data	Non-normality	Inability to contact patient	User workload
Underfitting	Proxy variables		Self-confidence
Overfitting	Implicit bias		Variation in social trust
Proxy Measure	Group attribution bias		
	Experimental bias		

The FDA is arguably the leading medical device regulator for providing guidance for device developers and manufacturers in their AI-enabled devices. While technically the FDA jurisdiction is limited to the United States, several other jurisdictions provide fast-track means for FDA-cleared or approved devices to be put on the market in their own countries. Most recently, the UK MHRA has announced plans for such a recognition route to enable FDA-cleared and approved devices to be sold in the UK.

A paper authored by employees at the FDA was recently published, focusing specifically on regulatory concepts and challenges for AI-enabled medical imaging devices.^28^ This article emphasizes how radiology has been a pioneer in adopting AI-enabled medical devices in a clinical environment, but also highlights how these devices “come with unique challenges” including the need for large and representative datasets, dealing with bias, understanding impact on clinical workflows, and maintaining safety and efficacy over time.

One key innovation from the FDA is the concept of “Predetermined Change Control Plans for Artificial Intelligence/Machine Learning-enabled Medical Devices”. This idea was proposed in the FDA “Artificial Intelligence/Machine Learning Software as a Medical Device Action Plan” in January 2021,^29^ and in 2023 a draft guidance was published^30^ that describes how this approach would be used to provide what the FDA describes as “a science-based approach to ensuring that AI/ML-enabled devices can be safely, effectively, and rapidly modified, updated, and improved in response to new data”. Predetermined change control plans do not provide for “continuous improvement” in the way many AI proponents argue for, but does provide a means by which manufacturers of AI-enabled medical devices can optionally submit with their 510k or de novo submission, a document which describes the sorts of change that can be made to the device without re-review by the FDA, including how risks are mitigated.

The FDA also published a “discussion document” in 2023 on the use of AI and Machine Learning in the development of drugs and biological.^6^ While this document is not focused on medical devices, and as a discussion document, is less formal than a Guidance document, it does give further insights into thinking within the FDA on the role of regulators in the application of AI in medical applications and raises the issues that need to be addressed in ensuring safe and effective use of AI tools. These are summarized in the categories listed in Table 5.

**Table 5.** Issues to be addressed in ensuring safe and effective use of AI tools.

**Human-led governance, accountability, and transparency**	
	Ensure adherence to legal and ethical values, where accountability and transparency are essential for the development of trustworthy AI
**Quality, reliability, and representativeness of data**	
	Bias
	Privacy and security
	Provenance of data
	Relevance
	Replicability
**Model development, performance, monitoring, and validation**	
	In balancing performance and explainability, it may be important to consider the complexity of the AI model

The UK MHRA, which is in the process of updating its medical device regulatory structure following the UK’s departure from the European Union, is also considering the implications of AI on medical device pathways. One possible route they are considering is the so-called “airlock process”,^31^ which provides a means to put some devices on the market with limited pre-market performance data:

*Some manufacturers of innovative products that meet a critical unmet clinical need may struggle to generate evidence in the premarket phase. Accordingly, this process will allow software to generate real world evidence for a limited period of time while being continuously monitored.*

This proposal is not yet implemented, and to have great value to device developers, it will need to link in with other international regulatory approaches.

In addition to taking account of publications from medical device regulators on AI-enabled devices, developers need to take account of other relevant regulations such as data privacy and the European Union Artificial Intelligence act, which rather like the FDA discussion paper referred to above, puts in place requirements for transparency and governance around AI.

## AI-enabled medical imaging devices on the market

As stated in the Introduction section, data published by the FDA^5^ show that more than three-quarters of AI-enabled medical devices that received marketing authorization up to the end of July 2023 are for applications in radiology, with cardiology applications such as arrhythmia detection from ECG being second largest application at 10% of devices. Two-thirds of the radiology devices received their marketing authorization in the three years between August 2020 and July 2023. A spreadsheet of these 350 AI-enabled radiology devices was downloaded from the FDA website, and sorted based on the FDA product code. The FDA product classification database was then used to cross-reference product code against the type of device, to determine whether the devices are hardware based (eg, image acquisition devices) or software only, and whether these product codes are specific to AI-enabled products. The FDA 510(K) and 513(f) de novo databases^32^^,^^33^ were then searched to find indications of use for selected devices to identify the intended radiological application, and how they are intended to fit into clinical workflows.

In Tables 6 and 7, we summarize the result of this analysis. For each type of device, we tabulate the number of such devices cleared or approved by the FDA, and the associated product code. Table 6 lists the 124 AI-enabled hardware devices. None of these types of devices are AI specific, but some manufacturers are incorporating AI in these devices to provide features such as automatic delineation of image features.

**Table 6.** AI-enabled hardware radiology devices cleared by FDA August 2021 to July 2024.

Type of device	Number	product code
Ultrasonic pulsed Doppler imaging system	28	IYN
Ultrasonic pulsed echo imaging system	1	IYO
Mobile X-ray system	1	IZL
Computed tomography X-ray system	38	JAK
Emission computed tomography	8	KPS
Magnet resonance diagnostic device	26	LNH
Stationary X-ray system	3	MQB
Densitometer, bone	1	KGI
Image-intensified fluoroscopic X-ray system	2	OWB
Optoacoustic imaging system	1	QNK
Medical charged-particle radiation therapy system	15	MUJ

**Table 7.** AI-enabled software radiology devices cleared by FDA August 2021 to July 2024.

Type of device	Number	Product code	AI specific	Definition summary
System, image processing, radiological	56	LLZ	N	*Image visualization, enhancement, and segmentation*
Lung CT computer-aided detection	2	OEB	N	To assist radiologists in the review of multi-slice CT of the chest and highlight potential nodules that the radiologist should review
Liver iron concentration imaging companion diagnostic for deferasirox	1	PCS	N	The determination of iron in the liver for any indication where an assessment of liver iron concentration is needed
Medical image analyser	9	MYN	N	Now mainly reclassified to Class II
Computer-assisted diagnostic software for lesions suspicious for cancer	5	POK	N	Assist users in characterizing lesions identified on acquired medical images
Radiological computer-assisted triage and notification software	26	QAS	Y	Aid in prioritization and triage of time-sensitive patient detection and diagnosis based on the analysis of medical images
Radiological computer-assisted prioritization software for lesions	19	QFM	Y	To aid in prioritization and triage of time-sensitive patient detection and diagnosis based on the analysis of medical images acquired from radiological signal acquisition systems
Radiological computer-assisted detection/diagnosis software for lesions suspicious for cancer	10	QDQ	Y	To aid in the detection, localization, and characterization of lesions suspicious for cancer on acquired medical images (eg, mammography, MR, CT, ultrasound, radiography)
Automated radiological image processing software	68	QIH	(Y)	Automated radiological image processing and analysis tools. Software implementing artificial intelligence including non-adaptive machine learning algorithms trained with clinical and/or artificial data
Radiological image processing software for radiation therapy	23	QKB	Y	Semi-automatic or fully automated radiological image processing and analysis tools for radiation therapy
Radiological computer-assisted detection/diagnosis software for fracture	4	QBS	Y	Aid in the detection, localization, and/or characterization of fracture on acquired medical images (eg, radiography, MR, CT)
Image acquisition and/or optimization guided by artificial intelligence	2	QJU	Y	Aid in the acquisition and/or optimization of images and/or diagnostic signals
Radiological machine learning-based quantitative imaging software with change control plan	1	QVD	Y	Software-only device which employs machine learning algorithms on radiological images to provide quantitative imaging outputs

Table 7 summarizes how the 226 AI-enabled software medical devices are broken down by type of device and product code. Most of these types of software devices, unlike the hardware devices in Table 6, have an associated definition in the FDA product code database, which is summarized in the right-hand column. For product code LLZ, which is older than the others, there is no such definition provided in the FDA product code database so we have provided one in italics for comparability. In addition, most of these product codes are specific to AI-enabled devices.

Tables 6 and 7 illustrate the wide variety of radiology hardware and software products that have recently been cleared or approved by the FDA. They cover a variety of imaging modalities and clinical applications, and some treatment devices.

A largest number of devices are for product codes QIH (Automated Radiological Image Processing Software) and LLZ (System, Image Processing, Radiological: the product code historically used for PACS workstations). These AI-enabled devices provide more sophisticated image segmentation, enhancement, manipulation, and visualization tools than traditional radiology PACS workstations.

The product code with the third greatest number of devices in Table 7 is QAS, Radiological Computer-Assisted Triage And Notification Software. Examination of the indication for use of these devices showed that 69% of these are for analysis of head CT for the purpose of triage of patients with acute brain injury (intracranial haemorrhage, stroke, brain trauma, large vessel occlusion), with most of the rest for use of chest CT for triage of patients with possible pulmonary embolism or aortic dissection. The indications for use of these devices, however, emphasize that they need to be used under the supervision of imaging experts, with statements such as: “not intended to be used as a primary diagnostic device”, “notified clinicians are ultimately responsible for reviewing full images per the standard of care”, and to be used “in parallel with standard of care”. This indicates how, for AI-enabled medical imaging devices, risks of inadequate performance currently need to be mitigated by ensuring significant clinical supervision.

While we are seeing increasing numbers of AI-enabled radiology devices coming onto the market, this analysis shows that the impact of these recently marketed devices on clinical practice is likely to be more incremental than disruptive, more as an adjunct to current radiological workflows, than significantly changing workflows.

## Discussion

There have been large numbers of publications on applications of AI and machine learning to medical imaging and radiology, and hundreds of medical devices placed on the market that are based on machine learning and AI tools. This rapid innovation, however, has highlighted some important challenges that the field needs to address in order for these innovative tools to be trusted by patients and healthcare professionals. In particular, there is increasing evidence that poorly implemented AI could lead to patient harm, and there is a need to identify and mitigate the underlying risks.

Two key challenges for the field are dealing with *bias* that might detrimentally impact real-world performance, and ensuring that the output is relevant to clinical care, that is, *clinically meaningful*. These challenges are illustrated by the role that publicly available datasets have played in catalysing innovation in AI algorithms. There is now a wide range of publicly available datasets that can be used to train machine learning image analysis algorithms, and here we will in particular consider the UK Biobank and the Alzheimer Disease Neuroimaging Initiative (ADNI). These datasets have driven a lot of high-quality science, but they do not include a representative sample of the general population, and illustrate the problem of bias in algorithms used to train imaging AI models.

### Bias

Petrick et al^28^ reported that a particular concern of regulators is how studies used to evaluate performance are “often based on limited patient, group and site diversity”, and it is not clear how these generalize to actual clinical practice.

Large publicly available datasets, such as the UK Biobank and ADNI dataset, are skewed in terms of demographics. Fry et al reported that “UK Biobank participants were more likely to be older, to be female, and to live in less socioeconomically deprived areas than nonparticipants”.^34^ Borchert et al^25^ undertook a systematic review in which they considered the role of ADNI in published algorithms using AI for diagnosis and prognosis in neurodegenerative disease. They reported that 71% of these algorithms rely on the ADNI data, which introduces multiple sources of bias. They go on to argue firstly “potential ethnic and socio-economic biases… that may hamper generalization”, and in addition that the image acquisition may be unrepresentative of current clinical data collection, introducing a bias related to the data. Similar issues with bias in training sets have been raised for AI-enabled computer-aided diagnosis (CAD) in mammography^35^ including unrepresentative patient populations and image acquisition protocols and vendors.

The skewed nature of these training datasets illustrates the importance of considering the sources of bias presented in Table 4 in the development and validation of AI-enabled medical devices, and the need to use independent training and test datasets, with the test datasets being representative of the intended population, to ensure that relevant performance data are obtained. While artificial data can be used as part of the assessment of AI-enabled device performance (eg, FDA product code QIH in Table 7), there is a risk that if generative AI is used to simulate large numbers of additional training or test data based on these biased datasets, this bias will be amplified.

### Clinical meaningfulness

The widespread availability of well-curated public databases has catalysed the innovation of AI tools, but a perverse consequence is that they encourage algorithm developers to focus on problems implicit in the datasets, rather than challenges in clinical care. For example, many authors developing algorithms trained on the ADNI dataset demonstrate that they can separate subjects who are “normal”, “mild cognitive impairment” (MCI), or “Alzheimer’s Disease” or that they can accurately predict conversion of MCI to early AD. However, not only do the patients enrolled in ADNI not represent the typical patient population in a community memory clinic, but these sorts of classifiers may not be relevant to addressing a clinically meaningful question. For example, if a patient arrives in a memory clinic with impaired memory, the question is not likely to be “does this patient have MCI or AD”, but “what is the underlying pathology causing these symptoms”, as that can impact subsequent management. Borchert et al reported that in their systematic review “We found no studies that assessed the common clinical challenge of differential diagnosis from among multiple (>2) possible diagnoses”, which is quite a strong critique of the field.

The regulatory framework for AI-enabled medical devices described in this article has relevance to addressing these sorts of limitations in academic AI tool development. The Clinical Evaluation SaMD framework helps clearly define the need to evaluate performance in the context of clinical care; Good Machine Learning Practice makes clear the importance of independent datasets for testing and validating (you should not use a single dataset like UK Biobank or ADNI for both training and testing), and the FDA recognized consensus standard AAMI CR34971:2022 provides a detailed framework for identifying and mitigating risks such as bias in AI-enabled devices.

## Conclusions

Artificial intelligence has already demonstrated it has great potential to enable novel and valuable medical technologies, and the great majority of AI-enabled medical devices marketed are for medical imaging applications. However, as the examples given earlier in this article illustrate, the literature contains many papers that justify the medical device regulators’ position that these methodologies introduce risks that are different, and in many cases greater, than the risks present in traditional “rule-based” software medical devices. As a consequence, AI-enabled devices on the market mitigate these risks with indications for use that require they be used under expert supervision, often in parallel with current clinical practice, reducing their likely impact on clinical practice.

For AI to have a greater clinical impact, developers of AI-enabled medical imaging tools need to provide more rigorous risk analysis and performance assessment than traditional software methods that are already on the market. Radiologists and their professional bodies have a key role to play in helping imaging AI researchers and device developers to put in place more rigorous frameworks for developing medical imaging AI devices, and monitoring their performance on the market in clinical practice. Radiologists and their radiographer and medical physics colleagues have a detailed understanding of the variation in patient presentation, impact of artefacts, variability due to radiographic practice, and variability caused by different imaging device manufacturers and acquisition parameters, which is of great value in helping identification and mitigation of risks in AI medical imaging tool development.

Also, as the technology evolves, the regulatory landscape is likely to continue to evolve, and in particular, ways in which AI software can be updated once on the market, and ways in which the balance of pre-market and post-market performance data can be used to demonstrate safety, are likely to evolve in the near future.

The evolving regulatory landscape can be criticized for providing developers with a “moving target” by rapidly changing the documentation required before AI-enabled medical devices can be put on the market, thus providing a barrier to innovation. However, it is also arguable that regulators are being agile in providing developers with increasing clarity on how to manage risk and assess the performance of these evolving technologies, so as to enable safe and effective AI-enabled medical devices to reach patients. As always with the regulation of medical devices, there is a balance to be struck between enabling innovation and ensuring patient safety, and AI will continue to challenge the existing regulatory frameworks. Academic researchers developing AI-enabled devices should also familiarize themselves with this regulatory framework to improve the quality of their publications, and facilitate the transition of research output into products that can positively impact patient management.
